# Determinants of physical activity and sedentary behaviour in university students: a qualitative study using focus group discussions

**DOI:** 10.1186/s12889-015-1553-4

**Published:** 2015-02-28

**Authors:** Tom Deliens, Benedicte Deforche, Ilse De Bourdeaudhuij, Peter Clarys

**Affiliations:** Department of Human Biometry and Biomechanics, Vrije Universiteit Brussel, Pleinlaan 2, 1050 Brussels, Belgium; Department of Movement and Sports Sciences, Ghent University, Watersportlaan 2, 9000 Ghent, Belgium

**Keywords:** Determinants, Physical activity, Sedentary behaviour, Energy expenditure, University students, Focus groups

## Abstract

**Background:**

College or university is a critical period regarding unhealthy changes in energy related behaviours in students. The first objective of this explorative study was to identify determinants of physical activity and sedentary behaviour in Belgian university students. Secondly, we aimed to collect ideas and recommendations to increase physical activity and decrease sedentary behaviours in university students.

**Methods:**

Using a semi-structured question guide, seven focus group discussions were conducted consisting of 17 male and 29 female university students from a variety of study disciplines, with a mean age of 20.7 ± 1.6 yrs. Using Nvivo9, an inductive thematic approach was used for data analysis.

**Results:**

Students reported that both physical and sedentary activities were influenced by individual factors (e.g. perceived enjoyment, self-discipline, time and convenience), their social networks (e.g. (lack of) parental control, modelling, social support), physical environment (e.g. availability and accessibility, travel time/distance, prices), and macro environment (e.g. media and advertising). Furthermore, the relationships between determinants and university students’ physical activity and sedentary behaviour seemed to be moderated by university characteristics, such as residency, university lifestyle, exams and academic pressure. Recommendations for future physical activity interventions include improving information strategies regarding on-campus sports activities, cheaper and/or more flexible sports subscriptions and formulas, including ‘sports time’ into the curricula, and providing university bicycles around campus. Students also believed that increasing students’ physical activity might decrease their sedentary behaviour at the same time.

**Conclusions:**

The recommendations and ideas discussed in this study may facilitate the development of effective and tailored (multilevel) intervention programs aiming to increase physical activity and decrease sedentary behaviours in university students.

## Background

The transition from secondary school to university is often accompanied by unhealthy behaviour changes such as decreasing physical activity and increasing sedentary behaviour [1,2]. According to Keating’s review [3], 40-50% of college students are physically inactive. A more recent study in Czech university students reported that only 9% met the criterion of 10,000 steps every day [4]. Concerning sedentary behaviour, a UK study revealed that university students spent eight hours per day on sedentary activities such as studying, watching television, gaming, computer activities, sitting and talking, shopping and hanging out [5].

Physical activity (including active transportation) and sedentary behaviour have an important influence on students’ weight and overall health [1,3]. A great body of literature points out that higher physical activity levels are associated with lower health risks (incl. overweight and obesity related diseases) [6,7]. There is also growing evidence that excessive participation in sedentary behaviours, such as watching television, computer use and sitting for work/study purposes, is associated with higher risk of obesity, independent of diet and physical activity behaviour [8-11]. Despite the fact that internet use as a specific sedentary behaviour may induce mental health benefits due to its use for social connection and support [10,12], research shows that higher levels of sedentary behaviour are associated with indicators of poorer well-being, increased risk of depression, and weaker cognitive functioning [10,12-14]. It is important to investigate physical activity and sedentary behaviour as two distinct modes of behaviour influencing weight and health independently [15]. Although Rouse et al. [5] concluded that physical activity and sedentary behaviours in university students appear largely uncorrelated, other studies showed a negative correlation between sedentary behaviours and physical activity in college students [16,17]. In order to prevent weight and fat gains in students, interventions should aim to increase physical activity and decrease sedentary behaviours, whether or not independently from each other [9].

Understanding why young people (do not) engage in physical activity and sedentary behaviour is important for intervention efforts encouraging more active lifestyles [18]. According to Swinburn et al. [19] individuals interact in a variety of micro-environments or settings (e.g. schools, workplaces, homes, (fast food) restaurants) which, in turn, are influenced by the macro-environments or sectors (e.g. food industry, government, society’s attitudes and beliefs). Ecological models consider the connections and the continuous interactions between people (intrapersonal) and their (sociocultural, policy and physical) environments [20-23].

In the literature, demographics (e.g. age, gender), psychological factors (e.g. self-efficacy, perceived enjoyment), social factors (social support from family and friends), and physical environmental factors (e.g. living/built environment, access to facilities) were reported to be possible influencing factors of college/university students’ physical activity (including active transportation) behaviour [3,24-26]. According to Haase et al. [24], physical activity is also related to some macro environmental factors, such as cultural factors and stage of national economic development. Although some studies investigated determinants of physical activity in university or college students, there is a lack of information on how to change these determinants and how to increase physical activity in this specific population. Asking participants what strategies may be effective to increase their physical activity may contribute to develop tailored and effective intervention programs. Moreover, the implementation of interventions based on ideas from students themselves may be more feasible and sustainable on a university campus. A participatory approach as such gives us an idea if and in what kind of strategy students are prepared/willing to participate. A review on factors affecting program implementation concluded that ‘shared decision-making’ (i.e. community involvement or participation) not only consistently led to better implementation, but also to higher sustainability of the program [27]. Thus, intervention programs taking the target group’s opinions into account may be more likely to succeed in the long run. Furthermore, there are no studies investigating determinants of sedentary behaviour in university or college students and no studies investigating possible intervention strategies. Previous studies investigating factors related to sedentary behaviours in young people were limited to TV viewing [28]. There is a need to examine determinants and correlates of total sedentary behaviour rather than TV viewing alone [28]. In addition, information on how to decrease sedentary behaviours in university students is lacking.

In summary, research in university students is still needed to better understand energy expenditure behaviours in order to develop effective strategies aiming to increase physical activity and decrease sedentary time [10]. Therefore, this study used a qualitative research design to explore which factors influence Belgian university students’ physical activity (including active transportation) and total sedentary behaviour (including screen behaviour, school work, socialising, and passive transportation). Furthermore, we collected ideas and recommendations in order to facilitate the development of tailored intervention programs aiming to increase physical activity and decrease sedentary behaviours in university students.

## Methods

### Participants

In this qualitative study focus group discussions were used for data collection. To ensure sufficient diversity of opinion, students from the second through the fifth year of university from different study disciplines were recruited using snowball sampling. Possible participants were approached face-to-face, by telephone or by email. No first year students were included because of their ‘limited’ experience as a university student. The aim was to recruit between six and ten participants per focus group [29].

### Procedure

Focus groups were held until saturation of new information was reached, as in qualitative research sample size can never be pre-determined [29]. To be sure we did not miss any ‘new’ information, one additional focus group session was held after theoretical saturation was estimated. All focus groups were organised in a conference room with an oval table at the Faculty of Physical Education and Physiotherapy of the Vrije Universiteit Brussel (Brussels, Belgium) at a time and date convenient for the students and researchers. Before each focus group all participants were asked to complete a short questionnaire, including demographics, height, weight and perceived health. Each focus group was facilitated by a moderator and an assistant moderator (observer). The moderator (male PhD student and first author of this manuscript) was trained and prepared through participating in workshops organised and guided by experienced focus group researchers. The assistant moderators (Master students, who were trained by the moderator) took notes during the discussions and made sure the moderator did not overlook any participants trying to add comments. Each focus group discussion started with an introductory round in which the researchers as well as the participants presented themselves and in which the researchers explained the purpose of the study. All focus group discussions were audiotaped with permission of the participants. Drinks and snacks were provided during the focus group discussions. Afterwards, all students received an incentive (a lunch voucher).

### Ethics statement

Before each focus group explanation about the aim of the study was given and an informed consent (in which participants’ anonymity and confidentiality was assured) was signed by each participant. The study was approved by the Medical Ethical Committee of the university hospital (Vrije Universiteit Brussel, Brussels, Belgium). All procedures followed were in accordance with the ethical standards of the responsible committee on human experimentation (institutional and national) and with the Helsinki Declaration of 1975, as revised in 2000.

### Question guide

According to recommended focus group methodology [30], a semi-structured question guide (see Table 1) was developed by the research team, aiming to identify factors influencing university students’ health and weight related behaviours (including eating (and drinking) behaviour, physical activity and sedentary behaviour). As mentioned, this paper will only focus on determinants of students’ physical activity and sedentary behaviour, as the results on determinants of students’ eating behaviour have already been published elsewhere [31]. After intensive collaboration with experts with ample focus group experience, the questions were carefully developed using appropriate literature [30]. When development was completed, the question guide was tested within and revised by the research team as well as pilot-tested in a group of ten university students. Because no major changes had to be made, ‘pilot’ discussion results were included in later analysis [29]. After some introductory questions, key questions focused on the main purpose of this study, i.e. identifying factors influencing students’ physical activity and sedentary behaviour. Finally, students were asked to share ideas concerning health promotion as well as intervention strategies to increase physical activity and decrease sedentary behaviour in university students. By using side questions, the moderator could guide the focus group discussions in the right direction and deviate the conversation when distracted from the main issue, or when participants were not talking about the appropriate behaviour. Because the pilot focus group discussion revealed that participants were not fully aware of the meaning of sedentary behaviour, a hand-out power point presentation explaining sedentary behaviour was given to all participants before the start of each focus group discussion. Sedentary behaviour was defined as activities requiring low levels of energy expenditure that occur while sitting or lying down [32]. In addition, following examples of sedentary activities were given: television watching, computer activities (screen time), classes, studying, socialising, and passive transportation. More detailed methodological information can be found in Deliens et al. [31].

**Table 1:** **Focus group question guide**

**Question type**	**Question**
Opening	1. Where are you from and what’s your name?
Introduction	2. Describe a healthy person.
Transition	3. Thinking of ‘health in university students’, what comes to your mind?
	4. Think back of the last year(s) being a university student. Did your body weight and/or body composition change since you entered university?
	5. Did your weight related behaviours (including eating, physical activity and sedentary behaviour) change since you entered university?
Key	6. Which factors have caused these changes? Which factors influence current weight related behaviours (including eating, physical activity and sedentary behaviour)? What barriers and enablers of weight related behaviour (including eating, physical activity and sedentary behaviour) can you identify?
	7. Which of the previous mentioned factors have had the greatest influence?
	8. Soon, we will try to help students make healthier choices. Can you give us some advice on how to promote healthy weight related behaviours (including eating, physical activity and sedentary behaviour) in students?
Ending	9. Do you have any remarks, suggestions, additions?

### Data analysis

SPSS Statistics 20 was used to analyse data obtained from the questionnaire and to calculate descriptive statistics of the focus group sample. Data obtained from the audio tapes where transcribed verbatim in Microsoft Word using Express Scribe and Windows Media Player. All quotes were encoded using the qualitative software program Nvivo9. Data were analysed using an inductive content analysis approach. In a first step, data (quotes) were examined for recurrent instances of some kind, which were then systematically identified across the data set, and grouped together by means of an open coding system (= content analysis) [33]. In a second and third step, themes were derived from the data, i.e. similar codes were grouped together into more general concepts (subcategories) and further categorised into main categories. This approach also allowed us to identify moderating factors that influence the strength of the relation between the determinant and the independent variable [34]. More detailed information about inductive content analysis can be found elsewhere [33,35]. To ensure reliability of coding and data interpretations, analyses were carried out independently by two researchers. Doubts or disagreements were discussed with two other researchers until consensus was reached.

## Results

In this study, the estimated point of saturation was observed after the sixth focus group session. One additional focus group discussion was conducted to be sure true saturation was established. In total, seven focus group discussions were conducted consisting of five to ten participants per group. The sample (n = 46) consisted of 17 male and 29 female students with a mean age of 20.7 ± 1.6 yrs (range = 18–26 yrs) and a mean study career of 3.2 ± 1.0 yrs. Each focus group discussion lasted between 90 and 120 minutes (including questions about eating behaviour which were not included in this paper). Additional sample characteristics are described in Table 2.

**Table 2:** **Characteristics of focus group participants (Mean ± SD, %, n = 46)**

Gender (% of females)	63.0
Age (yrs)	20.7 ± 1.6
Body mass index (BMI) (kg/m^2^)	22.4 ± 3.6
Underweight (%)	10.9
Normal weight (%)	67.4
Overweight (%)	21.8
Study career (yrs)	3.2 ± 1.0
Study discipline	
Human sciences (%)	67.4
Exact and applied sciences (%)	13.0
Biomedical sciences (%)	19.6
Residency (% living in student residence)	54.3
Smoking (% smokers)	8.7
Self-reported health (% reporting poor to very poor health status)	13.1
Perceived physical activity level (% reporting little to no physical activity)	54.3
Perceived eating pattern quality (% reporting poor to very poor eating pattern)	19.5

**Figure 1:**
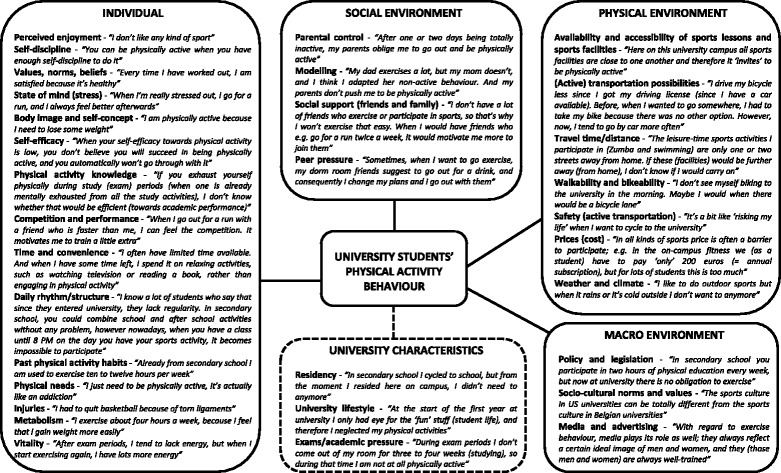
**Factors influencing physical activity behaviour of university students.**

**Figure 2:**
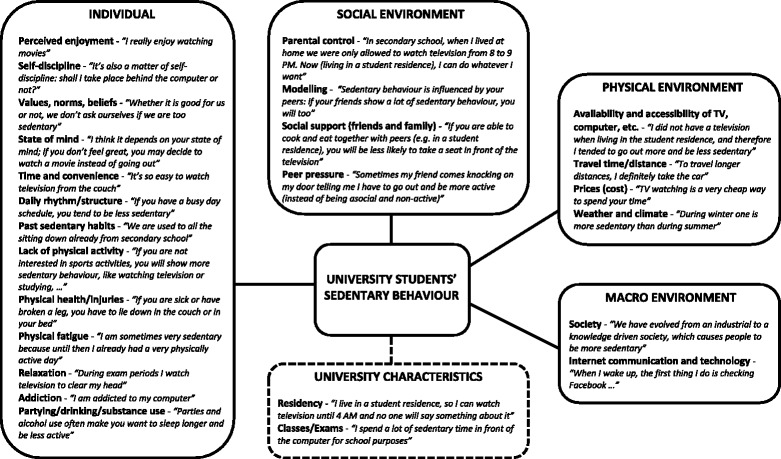
**Factors influencing sedentary behaviour of university students.**

According to the ecological principles two frameworks of factors influencing physical activity (Figure 1) and sedentary behaviour (Figure 2) in university students were developed based on content analysis of the focus group discussions. Both frameworks consist of four major levels, i.e. individual (intrapersonal), social environment (interpersonal), physical environment (community settings), macro environment, and an additional level of university characteristics. The most appropriate quotes were chosen to illustrate each (sub)category.

### Suggestions for physical activity and sedentary behaviour interventions

Students indicated that the university offers a lot of on-campus facilities and sports lessons, but at the same time they felt they were not well informed. Therefore, students suggested to improve communication and promotion strategies: *“I am sure there are lots of activities on campus, but unless you go ask and inform yourself, you don’t have a clue of what kind of activities are being offered”*. According to participants, first year university students could be informed during university’s open house days, or guided campus tours during the first week of university. They also suggested to promote all activities using media tools students are familiar with, such as Facebook. One student came up with the idea to organise a sports day (including initiation courses of all kinds of different sports) for all university students, giving them the possibility to get to know the university’s sports program and to subscribe themselves in one of the numerous sports activities. Students also explained that cheaper and/or more flexible sports subscriptions and formulas would lower the barriers of participation: *“One should offer cheaper sports activities and/or flexible subscriptions for 10 lessons giving you the freedom of choosing which lesson you want to participate in (e.g. swimming, Zumba, …)”*. Another student mentioned that such sports lessons should be accessible for beginners: *“if your condition is in bad shape, you should be able to join without any trouble”*. Other students suggested that some ‘sports time’ could be incorporated as part of the curricula: *“One could incorporate non-obligatory sports activities in students’ schedules. Also, it enables you to get to know your class mates, so it improves social cohesion as well”*. With regard to active transportation, students believed that providing ‘university bicycles’ (similar to the city bicycles available in large cities all around the world) could increase the use of bicycles for active transportation purposes: *“It would be great if you could subscribe into a bicycle rental program allowing you to rent a bicycle for a certain amount of time”*. Concerning sedentary behaviour, students believed that *“when making sports activities more easy accessible and more pleasant, students would spend less time on the couch”*. Participants also added that cultural activities (like exhibitions and museums) should therefore be promoted as well.

## Discussion

The purpose of this explorative study was to identify determinants of physical activity and sedentary behaviour in Belgian university students. Furthermore we collected ideas and recommendations in order to facilitate the development of tailored intervention programs aiming to increase physical activity and decrease sedentary behaviours in university students. Similar to Story’s framework [22] combining Bandura’s Social Cognitive Theory [36] with Sallis’ ecological model [21] explaining health behaviour, we identified four major levels of determinants: individual, social environment, physical environment and macro environment. In turn, these determinant levels were found to be influenced by some university specific characteristics.

### Individual

Many psychological factors such as perceived enjoyment, self-discipline, values, norms and beliefs, and time management were found to influence physical activity and sedentary behaviour at the same time. In Keating’s review [3] ‘having fun’ has been addressed as one of the primary reasons for college students to participate in physical activity or enrol in elective physical activity courses. With regard to time management, previous US studies using focus group discussions revealed that students feel like they lack time to be physically active [37-39]. Students spend a lot of time on study related sedentary activities (e.g. sitting in class, studying, or sitting in front of their computer for academic purposes), which makes it difficult to be physically active [37,38]. To counter time constraints, participants in the present study suggested to incorporate ‘sports time’ as part of their curriculum. With regard to time spent seated in classes, previous research has shown that taking a five minute walking break every hour could yield beneficial weight control or weight loss results [40]. Hence, it should be the task of university policy makers to integrate sufficient break-time during prolonged classes. Moreover, class schedules can be arranged in such a way that students have to relocate by foot or by bike between classes.

Due to the lack of interest in physical activity students often replace time they should ideally spend on physical activity with sedentary activities. On the other hand, when students are very physically active throughout a certain part of the day, physical fatigue might cause them to be more sedentary during the rest of the day. Although relaxation was not mentioned to be a reason to be physically active, students felt they rather needed to engage in sedentary activities (such as TV watching) to clear their heads. This might indicate that university students still choose sedentary over physical activities in terms of relaxation and recreation. Hence, physical activity promoters and policy makers are challenged to convince students to engage in physical activities for relaxation purposes. Finally, students revealed that there is an absorbing quality to some sedentary activities, such as spending time on social media. Therefore, the compulsive nature of certain sedentary behaviours, such as computer use (incl. social media access), should be taken into account with regard to intervention efforts.

Concerning future interventions, the present study’s findings support LaCaille’s [39] suggestion to strengthen students’ self-regulation skills (e.g. self-discipline, time management) around exercise as part of the transition from secondary school to university. McArthur and colleagues [41] showed that self-management strategies were strongly associated with physical activity level. Moreover, our results suggest that the same self-regulation skills should be addressed when aiming to decrease sedentary behaviour during this transition period. A randomised trial in college students showed that a 30-minute single session of one-on-one motivational interviewing (including discussing perceived benefits and barriers, personalised feedback, goal setting and strategies for increasing physical activity levels) increased moderate and vigorous physical activity levels after one month [42]. Although no long-term effects were evaluated, a single session intervention as such may be more appealing to college students and easier to implement on college campuses in comparison to more intensive models [42]. Maybe even more appealing to college students is the use of smartphone applications. Recent research in primary care patients showed that combined goal setting with an assisting smartphone application (based on self-monitoring and personalised feedback) significantly increased the amount of steps per day in comparison to a goal setting non-application control group [43]. Moreover, Bond and colleagues [44] showed that prompting small physical activity breaks after excessive sedentary time through a smartphone application increased physical activity and decreased sedentary time in overweight and obese individuals. To the best of our knowledge, no intervention efforts have been made so far to decrease sedentary behaviour in a university or college student population. Future (smartphone-based) experimental studies should investigate if similar motivational and behaviour change techniques are also effective in decreasing the amount of time university students spend in sedentary mode.

### Social environment

Although at the interpersonal level focus group literature in US university students only mentioned social support from friends to be influencing physical activity [37,39], the present study demonstrated that the social environment influencing students’ physical activity also included parental control, modelling and peer pressure. At the same time, these factors were found to influence students’ sedentary behaviour as well. The fact that previous US studies did not find parental influences on students’ physical activity behaviour might be explained by the longer home-university distances, forcing US university students to reside away from home more often than Belgian students, resulting in less parental influences.

### Physical environment

In accordance with previous US research investigating determinants of physical activity [37], current study results showed that university students are very susceptible to monetary costs. Moreover, results revealed that price can be a barrier to participate in healthy exercise behaviour, but at the same time be an enabler to choose other non-active or sedentary (like TV viewing) behaviour. Therefore, students proposed to make (on-campus) sports activities cheaper and/or more flexible which would lower the barriers of participation, resulting in opting for more physical and less sedentary activities.

Availability and accessibility of sports lessons and facilities as well as TV or computer were found to influence university students’ physical and sedentary activities. Despite the abundantly available on-campus sports facilities (at our university), participants of the present study did not automatically engage in more physical activities and/or less sedentary time. In Keating’s review [3] it was concluded that the influence of campus exercise or fitness facilities on university students’ physical activity behaviours was still unclear. The same review also revealed that research on the impact of campus size and overall physical layout and structure on physical activity has been neglected so far [3]. Hence, experimental research investigating the relative importance of physical environmental factors on physical activity, but also on sedentary behaviour in university students is needed. The abovementioned might also indicate that physical activity and sedentary behaviour in university students is not only influenced by the physical environment, but also the social environment and individual factors at the same time. The continuous interaction between determinant levels suggests that intervention strategies using multilevel approaches may be most effective [21].

### University specific characteristics

Some student characteristics (e.g. residency, exams, etc.) seemed to be moderating relationships between determinants and physical activity and sedentary behaviour. For example, living in a student residence might affect the strength of the relation between modelling and physical activity and/or sedentary behaviour. Students may experience less parental modelling but more peer modelling when residing away from home and vice versa.

### Physical (in)activity versus sedentary behaviour

Despite the introduction given on sedentary behaviour and its distinction from inactivity, it was hard to keep participants focused on sedentary behaviour as such. When asking them which factors influenced their sedentary behaviour, participants tended to deviate and talk about physically inactive behaviour instead. Therefore, the moderator had to be very alert and redirect discussions when necessary. Consequently, suggestions for interventions mainly focus on strategies to be more physically active, whereas little to no specific recommendations were made to target actual sedentary behaviour. This means that ‘sedentary behaviour’ is still a relatively unknown concept among university students, indicating that researchers along with policy makers still need to work on familiarising students with this concept and its association with overall health.

Although previous studies have shown that physical inactivity should be investigated independently from sedentary behaviour [5,15], many factors in the present study were found to influence physical activity and sedentary behaviour simultaneously. Students also believed that the lack of physical activity may increase the likelihood of spending more time in sedentary mode, suggesting an undeniable connection between both behaviours. Previous research in college students showed that computer use for men and television watching for women were negatively correlated with exercise and physical activity [16,17]. In accordance, Owen et al. [15] highlighted that sedentary behaviour can coexist with but also compete with physical activity. Therefore, as students suggested, intervention efforts aiming to increase university students’ physical activity might decrease time spent sedentary as well. It should be mentioned, however, that a recent review and meta-analysis of controlled trials in adults found that interventions aiming to promote physical activity (with no sedentary behaviour component) were least effective in reducing sedentary behaviour, compared to those studies that specifically targeted sedentary behaviour [45]. Thus, a component focusing on reducing sedentary behaviour may be needed to generate meaningful reductions in sedentary time [45].

### Strengths and limitations

This study adds important evidence to the limited literature investigating determinants of sedentary behaviour in university students and in general. Moreover, this is the first study collecting ideas and recommendations to increase physical activity and decrease sedentary behaviours in university students. This should facilitate the development of effective and tailored intervention programs aiming to improve physical activity and sedentary behaviour simultaneously. Secondly, as highlighted by Rouse et al. [5], sedentary behaviour is multifaceted and should not be limited to television viewing. Hence, participants of the present study were given a priori information on sedentary behaviour, making sure not only determinants of TV viewing but also other sedentary behaviours (e.g. computer use, studying, socialising) were explored. Finally, the research team chose focus group discussions over e.g. in-depth interviews, because the dynamic group interactions allowed us to get better insight into the mechanisms behind university students’ eating behaviours [33]. On the other hand, the group setting might have intimidated some participants which, in turn, might have limited a greater sharing of their thoughts.

Our study has some limitations as well. Although we might expect that behavioural differences according to gender may be found [28,46], we chose to use mixed-gender focus groups including students of different study years and disciplines, allowing us to create greater diversity of opinion within each focus group. Secondly, participants were recruited using snowball sampling, which is a purposive nonprobability approach that is often used in qualitative research, especially when the study is explorative in nature. This approach allowed us to generate rich and lively discussions, which may not happen in a more random collection of participants [29]. Using subjects who know one another may be a limitation to the generalizability of the findings beyond the group assessed. However, the purpose of this study was to generate a rich understanding of participants’ experiences and beliefs [47,48] and not to generalize results [49]. Finally, no quantification was used because the issue raised most frequently is not necessarily the most important, even when it is raised by a larger number of people [49]. In other words, each idea or opinion should be equally appreciated. Hence, future quantitative studies, using a larger representative sample, should determine the importance and value of each determinant, making differentiation according to gender or other student characteristics, as well as generalization possible.

## Conclusions

After entering university, students are continuously challenged by choosing between all kinds of activities (including physical and sedentary activities). Depending on their time schedule, a certain amount of self-discipline is needed to be physically active and not to engage in convenient sedentary activities. During this decision making process (= individual factors), students are influenced by their family and friends (= social environment), as well as by the availability and accessibility and prices of these activities (= physical environment). In turn, choices have to be made within a university specific setting, such as living in a student residence, having exams (= moderators). Recommendations for future physical activity interventions include improving information strategies regarding on-campus sports activities, cheaper and/or more flexible sports subscriptions and formulas, including ‘sports time’ into the curricula, and providing university bicycles around campus. Finally, students felt that the abovementioned recommendations to increase physical activity may decrease students’ sedentary behaviour at the same time. Our results should be considered a first step towards the development of tailored and effective intervention programs aiming to improve university students’ energy expenditure behaviours.

## Abbreviations

BMI: Body mass index
SD: Standard deviation
SPSS: Statistical package for the social sciences
TV: Television
UK: United Kingdom
US: United States
yrs: Years
